# Natural products in synthesis and biosynthesis

**DOI:** 10.3762/bjoc.9.223

**Published:** 2013-09-19

**Authors:** Jeroen S Dickschat

**Affiliations:** 1 Institut für Organische Chemie, Technische Universität Carolo-Wilhelmina zu Braunschweig, Hagenring 30, D-38106 Braunschweig, Germany

**Keywords:** natural products

This is the second Thematic Series in the Beilstein Journal of Organic Chemistry focused on natural products. While the first Thematic Series summarized recent research work on the biosynthesis and function of natural products [1–2], the present Thematic Series deals with synthetic and biosynthetic aspects.

**Figure 1:**
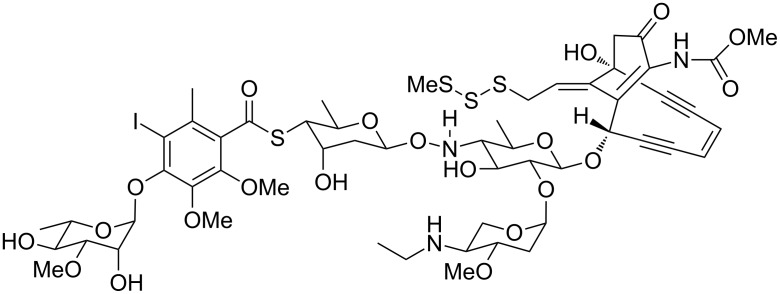
Calicheamycin.

Why the research interest in natural products? Natural products are structurally fascinating as is spectacularly demonstrated by the prominent examples of maitotoxin [3], the largest non-polymer secondary metabolite known to date, or calicheamycin (Figure 1), which possibly holds the record in carrying the most diverse functional groups including an enediyne subunit, a deoxysugar, an aminosugar, a hydroxylamine linkage between two sugar subunits, a carbamate, an aromatic iodide, a thioester, and a trisulfide [4]. Natural products have also been used by humankind since ancient times and are part of our traditions and culture, as is evident from the widespread consumption of coffee, tea, tobacco and cocoa, drugs that all contain alkaloids with stimulating effects. More importantly, starting from their usage in traditional medicine natural products – and today also compounds inspired by natural products – are indispensable in the treatment of various threatening infectious diseases. The rapid spreading of antibiotic resistances and the comeback of some infectious diseases already claimed to be eliminated should sharpen our minds for the beneficial roles of natural products in medicine as well as their responsible use. This should be a strong appeal upon the responsibility of both researchers and decision makers in the academic sciences and the pharmaceutical industry.

Why should we have a look at the synthetic and biosynthetic aspects of natural products in this Thematic Series? Of course, the chemistry of nature and as it is used by chemists is one and the same and makes use of the same intrinsic reactivity of molecules. It is fascinating to see that a particular chemical reaction, believed to be invented by man, is in fact already used for millions – sometimes even billions – of years by Mother Nature. An interesting example is the discovery of the Diels–Alder reaction in the 1920s and its later enhancement into an enantioselective reaction by the development of chiral catalysts. But a Diels–Alder reaction also occurs in the biosynthesis of lovastatin and is likely catalysed by a Diels–Alderase [5], a natural analogue of man-made chiral catalysts! Beyond such fascinating examples of comparing natural to artificial systems it is their interplay that may provide the best solutions to some of the most urgent problems of our time. A perfect example is artemisinin, a terpenoid natural product from *Artemisia annua*, which is highly efficient in the treatment of malaria. The problem is that the plant produces only small and variable amounts, thereby making the isolation procedure difficult and expensive. Neither biotechnology nor chemistry were able to provide a solution on their own, but the combination of the heterologous production of an advanced intermediate in yeast [6] and its further elaboration by chemical synthesis [7] procures sufficient quantities of artemisinin at a reasonable price. An impressive demonstration of the effectiveness of multi-disciplinary research.

I would like to thank the highly professional team of the Beilstein-Institut for a very pleasant cooperation on this Thematic Series. I wish you, the reader, joy and fun with this Thematic Series and – at the best – a new impetus for your own future research!

Jeroen S. Dickschat

Braunschweig, September 2013
